# Adiponectin inhibits the activation of lung fibroblasts and pulmonary fibrosis by regulating the nuclear factor kappa B (NF-κB) pathway

**DOI:** 10.1080/21655979.2022.2063652

**Published:** 2022-04-17

**Authors:** Xin Wang, Jian Yang, Liangquan Wu, Chunran Tong, Ying Zhu, Wei Cai, Bing Wan, Xiuwei Zhang

**Affiliations:** Department of Respiratory and Critical Care Medicine, The Affiliated Jiangning Hospital with Nanjing Medical University, Nanjing, Jiangsu, P.R. China

**Keywords:** Adiponectin, pulmonary fibrosis, bleomycin, TGF-β1, NF-κB pathway

## Abstract

Idiopathic pulmonary fibrosis (IPF) is a common pulmonary interstitial disease with a high mortality rate. Adiponectin (APN) is reportedly an effective therapy for fibrosis-related diseases. This study aimed to investigate the potential effects of APN on IPF. Male BALB/c mice were injected with bleomycin (BLM) and treated with different doses of APN (0.1, 0.25, and 0.5 mg/kg). The body weights of the mice were recorded. Immunohistochemical, hematoxylin and eosin, and Masson staining were performed to evaluate pulmonary histopathological changes. Enzyme-linked immunosorbent assay (ELISA) and western blotting were performed to assess tissue inflammation. The human lung fibroblasts HELF were stimulated with TGF-β1 and treated with different doses of APN (2.5, 5, and 10 μg/ml). Cell proliferation, inflammation, and fibrosis were determined by MTT assay, EdU assay, colony formation assay, ELISA, and western blotting. APN significantly attenuated BLM-induced body weight loss, alveolar destruction, and collagen fiber accumulation in mice (*p* < 0.05). APN decreased the expression of α-SMA and collagen I and reduced the concentration of TNF-α, IL-6, IL-1β, and IL-18 in lung tissues (*p* < 0.05). In TGF-β1-treated HELF cells, cell proliferation and colony formation were inhibited by APN (*p* < 0.05). Additionally, the expression of α-SMA, collagen I, and pro-inflammatory cytokines were suppressed by APN (*p* < 0.05). APN inhibited the phosphorylation of IκB and nuclear translocation of p65. In conclusion, these findings suggest that APN is an effective agent for controlling IPF progression. The antifibrotic effects of APN might be mediated via inhibiting the NF-κB signaling pathway.

## Graphical Abstract

**Figure:**
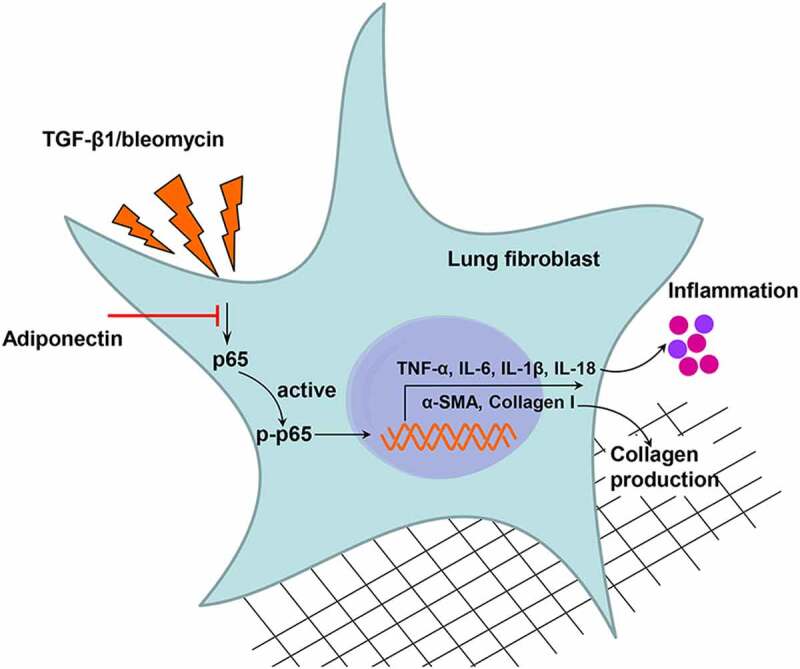

## Highlights

1. Adiponectin inhibits bleomycin-induced pulmonary fibrosis in mice;
2. Adiponectin inhibits TGF-β1-induced lung fibroblast activation;
3. Adiponectin inhibits TGF-β1-induced lung fibroblast inflammation;
4. Adiponectin inhibits the activation of NF-κB signaling.

## Introduction

Idiopathic pulmonary fibrosis (IPF) is the most common pulmonary interstitial disease that can occur in various clinical settings. As a type of age-related disease, morbidity among individuals older than 50 years is high, and its prevalence has increased steadily over time[1]. A progressive decline in lung function is the most typical clinical characteristic. The destruction of lung tissue results in respiratory distress, cough, and body weight loss, ultimately leading to death [2]. It has been reported that the survival of patients with IPF is approximately 3 years, which is shorter than that of patients with most types of cancers [3]. Currently, antifibrotic therapies, such as pirfenidone and nintedanib, are used clinically and are effective in treating IPF [4]. However, the response to pirfenidone and nintedanib is heterogeneous and limited by side effects, like diarrhea, nausea, diarrhea, and fatigue [4]. Therefore, novel antifibrotic therapies are required to be investigated to prolong the survival of patients with IPF.

Adiponectin (APN), also known as Acrp30, apM1, AdipoQ, and GBP28, are endogenous bioactive proteins secreted by adipocytes [5]. As a hormone, its main function is to regulate metabolism and maintain the body’s energy homeostasis [6]. Over the past two decades, the critical roles of APN in preventing a wide variety of diseases have been identified, such as diabetes [7], obesity, Alzheimer’s disease [8], chronic kidney disease [9], rheumatoid arthritis [10], and cancer [11,12]. It exerts strong protective roles in target organs, including kidney, brain, heart, skeleton, and lung, against cell apoptosis and inflammation induced by various stimulations [13]. APN level has been suggested as a significant prognostic factor in patients with IPF [14,15]. Previous studies performed in mice demonstrated that APN is a potential antifibrotic therapy for paraquat-induced pulmonary fibrosis [16,17]. However, the effects of APN on IPF have not been exhaustively revealed, and the underlying mechanisms are not completely understood.

Nuclear factor kappa B (NF-κB) is a pleiotropic transcription factor consisting of five family members: p65/RelA, RelB, cRel, p50, and p52. The five protein monomers form homodimers or heterodimers to bind with DNA and regulate different biological processes, including cell inflammation, immune response, and cell death [18]. The NF-κB signaling pathway plays a regulatory role in interstitial fibrosis [19], renal fibrosis [20], hepatic fibrosis [21], and lung fibrosis [22]. Inhibiting the activation of NF-κB signaling may be an effective strategy for treating lung fibrosis. In addition, previous studies have revealed that NF-κB pathway is one of the target signaling pathways of APN [23,24]. APN is capable of treating paraquat-induced pulmonary fibrosis, possibly by regulating NF-κB signaling [17]. However, the crosstalk between APN and NF-κB signaling in bleomycin (BLM)-induced IPF has not been investigated but has gained attention since BLM has been considered as the most important and widely used method for inducing lung fibrosis in animal models.

In this study, an animal model of IPF was established by injecting BALB/c mice with BLM. Meanwhile, a cell model of IPF was established by stimulating human lung fibroblasts (HELF) with TGF-β1, a factor that induces fibrosis and immunity [25]. Considering the fundamental role of TGF-β1 in the pathogenesis of fibrotic diseases, it has been widely used to construct a cell model of IPF [26,27]. Herein, the effects of APN were studied both *in vitro* and *in vivo*. The results of this study will broaden our understanding of the role of APN and indicate a novel antifibrotic therapy for IPF.

## Materials and methods

### Animals and treatment

SPF grade male BALB/c mice (6–8 weeks, 18–22 g) were purchased from Beijing Vital River Laboratory Animal Technology Co., Ltd. (Beijing, China) and housed conventionally in an SPF environment as previously reported [16]. The mice were randomly divided into five groups (n = 5/group): Control, BLM, BLM+APN (0.1 mg/kg), BLM+APN (0.25 mg/kg), and BLM+APN (0.5 mg/kg). BLM (1.5 U/kg; cat. No. 0219030610, MP Biomedicals, Santa Ana, CA, USA) was intraperitoneally injected into the mice [28]. Mice in the control group were injected with the same volume of saline. After BLM exposure, the mice were injected with different concentrations of APN (0.1, 0.25, and 0.5 mg/kg; cat. No. A04323, Shanghai Hewu Biological Technology Co., Ltd, Shanghai, China) via the tail vein [16]. The body weight of the mice was recorded every 3 days until sacrifice by cervical dislocation. The animal studies were approved by the Ethics Committee of our institution (ethical no. DW20191203), and all procedures were conducted in accordance with the standards of the National Institutes of Health Guide for the Care and Use of Laboratory Animals.

### Histopathological assessment of lung tissue

On the 21st day after APN injection, lung tissues were collected from each group, embedded in paraffin, and cut into 4 μm-thick sections. For immunohistochemistry, the sections were incubated with primary antibodies against α-SMA (1:100, cat. No. MBS8813489, MyBioSource, San Diego, CA, USA) and collagen I (1:50, cat. No. LS‑C829132, LifeSpan BioSciences, Seattle, WA, USA) overnight at 4°C. The images were captured using a microscope (Model DM6000B; Leica Microsystems GmbH, Wetzlar, Germany). Image-Pro Plus 6.0 software (Media Cybernetics, Rockville, MD, USA) was used to analyze the target protein displaying cytoplasmic or nuclear staining patterns. The staining intensity was scored as follows: 0 = no staining; 1 = weak; 2 = moderate; and 3 = strong. The extent of staining was defined as the percentage of the positive staining areas: 0 = 0% positive area; 1 = less than 25% of positive area; 2 = the positive area ranging from 25% to 50%; 3 = more than 50% positive area. The total score (range: 0–9) was calculated by multiplying the intensity and positivity scores. For hematoxylin and eosin (H&E) staining and Masson staining, the injury area and Ashcroft score were the pathology indices [16]. Briefly, the pathology index of H&E staining was calculated according to the following scale: 0, no injury; 1, 25% of injury field; 2, 50% of injury field; 3, 75% of injury field; and 4, 100% of injury field. The fibrotic area stained by Masson was quantified according to the following scoring system: 0, normal lung; 1, minimal fibrous thickening of alveolar or bronchiolar walls; 2, moderate thickening of walls without obvious damage to lung architecture; 3, increased fibrosis with definite damage to lung structure and formation of fibrous bands or small fibrous masses; 4, severe distortion of the structure and large fibrous areas; and 5, total fibrosis. Ten high-power fields (400×) were selected for use in the calculation, and the score calculation was quantified by two histopathologists blinded to the protocol.

### Cell culture

HELF cells were purchased from the Cell Bank of the Chinese Academy of Sciences (cat. No. GNHu28; Shanghai, China) and cultured in Dulbecco’s Modified Eagle’s Medium culture medium (cat. No. A4192101, Gibco) supplemented with 10% fetal bovine serum (cat. No. 10099141, Gibco). The cells were maintained in 5% CO_2_ atmosphere at 37°C. HELF cells at passage 4 to 8 were used in the following experiments. The number of HELF cells in a suspension is calculated using a hemocytometer chamber [29]. HELF cells were treated with 5 ng/ml TGF-β1 (cat. No. RP-39357, Invitrogen, Carlsbad, CA, USA) for 24 h ^30^and then treated with different doses of APN (2.5, 5, and 10 μg/ml) for 48 h at 37°C [16]. Bay 11–7082 (cat. No. HY-13453, MedChemExpress, Monmouth Junction, NJ, USA) at a concentration of 5 μM was added to cells for 1 h to inhibit the activation of NF-κB signaling pathway [30].

### MTT assay

HELF cells were subjected to TGF-β1 and APN treatments followed by 3-(4, 5-dimethylthiazol-2-yl)-2, 5-diphenyltetrazolium bromide (MTT) analysis. Cells with a density of 5 × 10^3^ cells/well were seeded in 96-well plates and were stained with 0.5 mg/ml MTT solution (ab211091, Abcam, Cambridge, MA, USA) for 4 h at 25 ± 2°C. After incubation with 150 μl dimethyl sulfoxide (cat. No. 94,563, Sigma-Aldrich) to dissolve formazan, the absorbance was measured using a microplate reader (Model 550; Bio-Rad, Hercules, CA, USA).

### EdU incorporation assay

The proliferation of HELF cells was determined using the 5-ethynyl-2-deoxyuridine (EdU) assay kit (cat. No. C10310-1; Ribobio, Guangzhou, China). The incorporation of EdU into cellular DNA and the reaction of EdU with a fluorescent azide are useful for quantification of DNA synthesis during replication. Briefly, cells in 6-well plates were incubated with 50 μM EdU for 2 h and then fixed with 4% formaldehyde for 30 min at 25 ± 2°C. Triton X-100 (cat. No. 93,443, Sigma-Aldrich) at a concentration of 0.5% was used to increase the membrane permeability. After washing the cells with PBS three times, 4’,6’-diamidino-2-phenylindole (DAPI; cat. No. D9542, Sigma-Aldrich) was added for 30 min to stain the nuclei. The stained cells were photographed under a fluorescence microscope (IX73; Olympus, Tokyo, Japan), and the number of EdU-positive cells was counted using the Image-Pro Plus 6.0 software.

### Colony formation assay

HELF cells were planted in 6 cm culture dishes, treated with TGF-β1 and APN and incubated for 8 days. Then, colonies were fixed with absolute ethyl alcohol and stained with 1% crystal violet (cat. No. C6158, Sigma-Aldrich) for 10 min at 25 ± 2°C. Methanol was added and the absorbance at 540 nm was recorded using a microplate reader (Model 550; Bio-Rad).

### Enzyme-linked immunosorbent assay (ELISA)

The levels of IL-6, TNF-α, IL-1β, and IL-18 in lung tissues and the culture supernatants of HELF cells following the indicated treatment were measured using ELISA kits (cat. No. KE00139, KE00154, KE00021, KE00193, respectively; Proteintech, Chicago, IL, USA), in accordance with the manufacturer’s instructions. The kit was assessed using the criteria of parallelism to a standard curve.

### Quantitative real-time polymerase chain reaction (qRT-PCR)

Total RNA from lung tissues and HELF cells was extracted using the RNAiso Plus (cat. No. 9109, TaKaRa Biotechnology, Dalian, China) in a sterile environment. The concentration and purity of the isolated RNA were determined by measuring the absorbance at 260 and 280 nm. qPCR was performed using the One Step TB Green® PrimeScript™ RT-PCR Kit (cat. No. RR066A, TaKaRa) and the StepOnePlus™ Real-Time PCR System (cat. No. 4,376,600, Thermo Fisher Scientific, Waltham, MA, USA). The primer sequences used in the study were designed and synthesized by GenePharma (Shanghai, China). The primer sequences used for qRT-PCR are listed in Table 1.

**Table 1.** Primer sequences used in qRT-PCR

Gene name	Forward	Reverse
Human α-SMA	5′-TATCCCCGGGACTAAGACGG −3′	5′-CACCATCACCCCCTGATGTC-3′
Human collagen I	5′-GAACGCGTGTCATCCCTTGT-3′	5′-GAACGAGGTAGTCTTTCAGCAACA-3′
Human GAPDH	5′-GTTGCAACCGGGAAGGAAAT-3′	5′-GCCCAATACGACCAAATCAGA-3′

### Western blot

Total proteins in lung tissues and HELF cells were isolated by using a Protein Extraction Kit (cat. No. KGBSP002, KeyGen Biotech Co., Ltd., Nanjing, China). Nuclear lysates were extracted using a Nuclear Extract Kit (cat. No. 40010, Active Motif, Shanghai, China). A bicinchoninic acid (BCA) protein assay kit (cat. No. MPK002, Macgene Technology Co., Ltd., Beijing, China) was used to assess the purity of the extracted lysates. After proteins were separated by sodium dodecyl sulfate-polyacrylamide gel electrophoresis and transferred onto PVDF membranes (cat. No. 03010040001, Sigma-Aldrich), the membranes were blocked in western blocking reagent (WESTBL-RO, Sigma-Aldrich) at 25 ± 2°C for 1 h. The membranes were incubated with primary antibodies at 4°C overnight. Primary antibodies against collagen I (1:3000, LS‑C829132), p65 (1:500, LS-C413576), p21 (1:200, LS-C136937), p27 (1:500, LS-C389941), TNF-α (1:500, LS-C18838), IL-1β (1:500, LS-C104781), and IκB (1:500, LS-C331335) were purchased from LifeSpan BioSciences. Antibodies against α-SMA (1:1000, MBS8813489) and IL-6 (1:1000, MBS2106139) were purchased from MyBioSource. Antibodies against PCNA (1:1000, ab18197), p65 (phosphor S529; 1:500, ab97726), IL-18 (1:1000, ab207323), Histone H3 (1:1000, ab1791), and GAPDH (1:2500, ab9485) were purchased from Abcam and antibodies against p-IκB (phospho Ser32/Ser36; 1:1000, GTX25682) were purchased from GeneTex (Irvine, CA, USA). GAPDH served as an internal control for proteins in whole-cell lysates. Histone H3 acted as an internal control for nuclear p65. After incubation with goat anti-rabbit (1:2000, A32731) or goat anti-mouse (1:2000, A32723) secondary antibodies (Thermo Fisher Scientific) for 1 h, enhanced chemiluminescence was performed to visualize the blots. The gray level of the target bands was analyzed using the Image-Pro Plus 6.0 software.

### Immunofluorescence staining

HELF cells were treated with TGF-β1 and APN and then immunofluorescence microscopy was performed to analyze p65 expression in cells. The cells were subjected to antigen retrieval and then incubated with a primary antibody against p65 (1:200, LS-C413576, LifeSpan BioSciences) at 25 ± 2°C for 2 h. After washing with Tris-HCl buffered saline with 0.1% (v/v) Tween 20, the cells were incubated with a secondary antibody (1:500, A32723, Thermo Fisher Scientific) for 1 h. A fluorescence microscope (IX73; Olympus, Tokyo, Japan) was used to obtain immunofluorescence images [31].

### Statistical analysis

All data are expressed as mean ± standard deviation (SD) from at least three independent experiments. Statistical differences among groups were analyzed using one-way ANOVA following Tukey post-hoc test using the SPSS 19.0 software (SPSS Inc., Chicago, IL, USA). For IHC scores, pathology index and Ashcroft scores, data were analyzed using Kruskal-Wallis with Dunn multiple comparisons test. A *p*-value less than 0.05 indicated a significant difference.

## Results

### APN ameliorates BLM-induced pulmonary fibrosis in vivo

**Figure 1.**
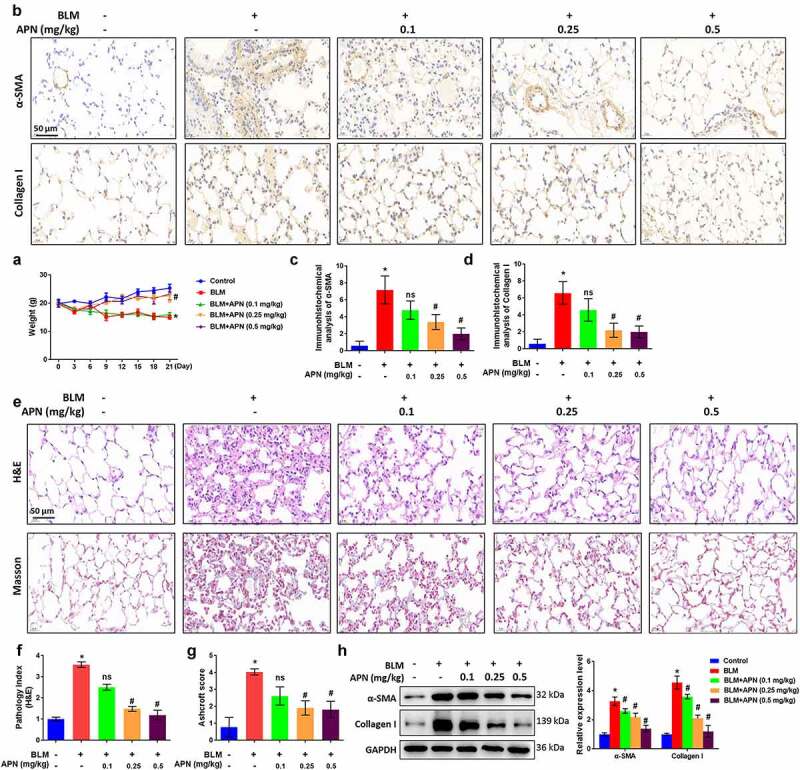
*In vivo* effect of APN on BLM-induced pulmonary fibrosis. BALB/c mice were injected with BLM and different concentrations of APN. (a) The body weights of mice were recorded every three days until sacrifice. (b) Immunohistochemical analysis results of α-SMA and collagen I in lung tissues and representative images were shown. Quantitative levels of (c) α-SMA and (d) collagen I. (e) H&E and Masson staining were conducted to assess the pathological injury and fibroblastic foci. Representative images were shown. (f) Pathology index from H&E staining and (g) Ashcroft score from Masson staining were shown. (h) Protein levels of α-SMA and collagen I in the lung tissues were analyzed by western blotting. n = 5. * *p* < 0.05 vs. nontreated group. ns, no significant, # *p* < 0.05 vs. BLM-treated group.

At first, we studied the effects of APN on pulmonary fibrosis and injury in BLM-treated mice. The body weights of mice treated with BLM and APN were recorded every 3 days until sacrifice. The results in Figure 1a indicate that BLM treatment caused a significant body weight loss, while high doses of APN (0.25 and 0.5 mg/kg) alleviated the weight loss (*p* < 0.05). Immunohistochemistry was performed to detect the effects of APN on the expression of fibrosis-related factors in the lung tissues. Figure 1b-d demonstrates that BLM significantly elevated the expression of α-SMA and collagen I (*p* < 0.05). The expression of α-SMA and collagen I induced by BLM was decreased by APN in a dose-dependent manner (*p* < 0.05). Next, H&E and Masson staining were performed to determine the histological condition of the lung tissues. BLM largely destroyed the alveolar structure and caused a remarkable collagen fiber accumulation (*p* < 0.05, Figure 1e-g). BLM-induced histological changes in pulmonary tissue induced by BLM were attenuated by APN treatment with a dose-dependent manner (*p* < 0.05). Unsurprisingly, western blot results showed α-SMA and collagen I upregulation in BLM-treated mice (*p* < 0.05, Figure 1h). APN inhibited BLM-induced expression of α-SMA and collagen I in a dose-dependent manner (*p* < 0.05). These results provide *in vivo* evidence that APN can inhibit BLM-related pulmonary fibrosis.

### APN ameliorates BLM-induced inflammation in vivo

**Figure 2.**
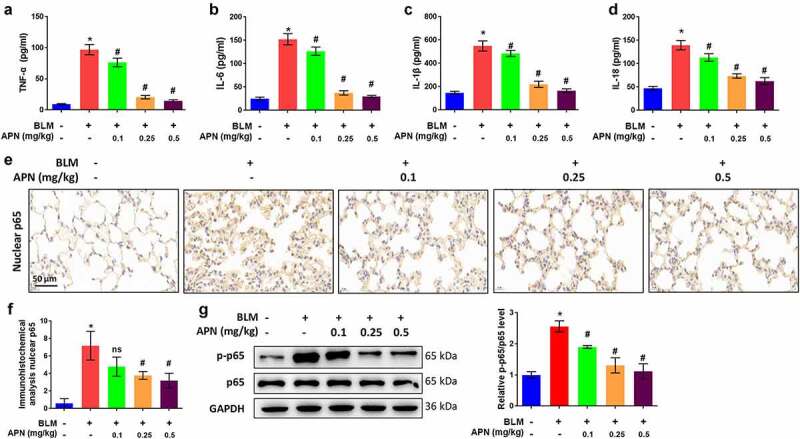
*In vivo* effects of APN on pulmonary inflammation. BALB/c mice were injected with BLM and different concentrations of APN. The concentrations of (a) TNF-α, (b) IL-6, (c) IL-1β, and (d) IL-18 in the lung tissues were measured by ELISA. (e) Immunohistochemical analysis of p65 and representative images were shown. (f) Quantitative level of nuclear p65 from immunohistochemical analysis. (g) The expression of p65 and its phosphorylation were measured by western blotting. n = 5. * *p* < 0.05 vs. nontreated group. ns, no significant, # *p* < 0.05 vs. BLM-treated group.

Next, the effect of APN on pulmonary tissue inflammation was investigated in BLM-treated mice. Systemic inflammation is typically measured by assessing the production and release of pro-inflammatory cytokines, including TNF-α, IL-6, and IL-1β. In addition, there is an increasing interest in the assessment of IL-18 in pulmonary fibrosis [32,33]. BLM remarkably elevated the concentrations of TNF-α, IL-6, IL-1β, and IL-18 in lung tissues (*p* < 0.05, Figure 2a-d). The BLM-induced release of these pro-inflammatory cytokines was attenuated by APN treatment in a dose-dependent manner (*p* < 0.05), indicating the anti-inflammatory function of APN. To reveal the molecular mechanism involved, we investigated the activation of NF-κB, which has been widely considered as a prototypical proinflammatory signaling pathway [34]. Immunohistochemistry results in Figure 2e, 2f revealed that BLM promoted the accumulation of p65 in the nuclei, while APN inhibited p65 accumulation (*p* < 0.05). Additionally, BLM promoted the phosphorylation of p65, whereas APN inhibited p65 phosphorylation (*p* < 0.05, Figure 2g). The anti-inflammatory function of APN in mice may be related to the NF-κB signaling pathway.

### APN inhibits TGF-β1-induced HELF cell proliferation

**Figure 3.**
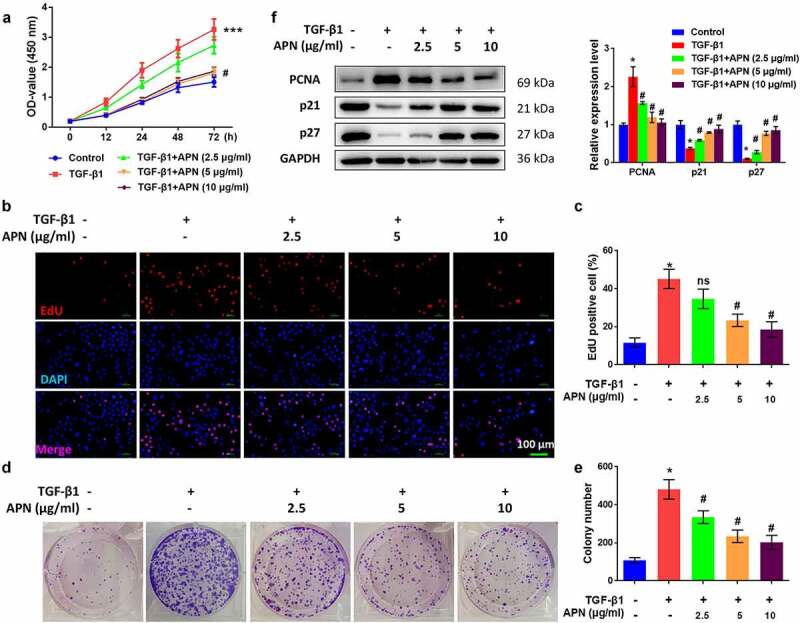
Effects of APN on HELF cell proliferation. HELF cells were treated with TGF-β1 and different concentrations of APN. Cell proliferation was measured by (a) MTT and (b) EdU assays. (c) Quantitative results of cell proliferation. (d) Colony formation of cells was measured and representative images were shown. (e) Quantitative results of colonies. (f) Expression of proliferation-related proteins was analyzed by western blotting. n = 3. * *p* < 0.05 vs. nontreated group. # *p* < 0.05 vs. TGF-β1-treated group.

To confirm the antifibrotic effect of APN *in vitro*, HELF cells were treated with TGF-β1 and then cell proliferation was tested. As shown in Figure 3a, TGF-β1 significantly enhanced the absorbance (OD) value of the MTT assay, while high doses of APN reduced the OD value (*p* < 0.05). The effects of APN on HELF cell proliferation were also confirmed by the EdU assay. TGF-β1 promoted the proliferation of HELF cells, while APN inhibited cell proliferation in a dose-dependent manner (*p* < 0.05, Figure 3b,c). Moreover, colony formation of TGF-β1-treated cells was promoted, while that of APN-treated cells was inhibited (*p* < 0.05, Figure 3d,e). Changes in the expression of cell cycle-specific proteins, including PCNA, p21, and p27, were analyzed to further confirm the role of APN in the regulation of HELF cell proliferation. TGF-β1 remarkably upregulated PCNA protein expression and downregulated p21 and p27 protein expression (*p* < 0.05, figure 3f). APN affected these three proteins in an opposite manner, indicating its anti-proliferative role in HELF cells via regulation of cell cycle-specific proteins.

### APN inhibits TGF-β1-induced fibrosis and HELF cell inflammation

**Figure 4.**
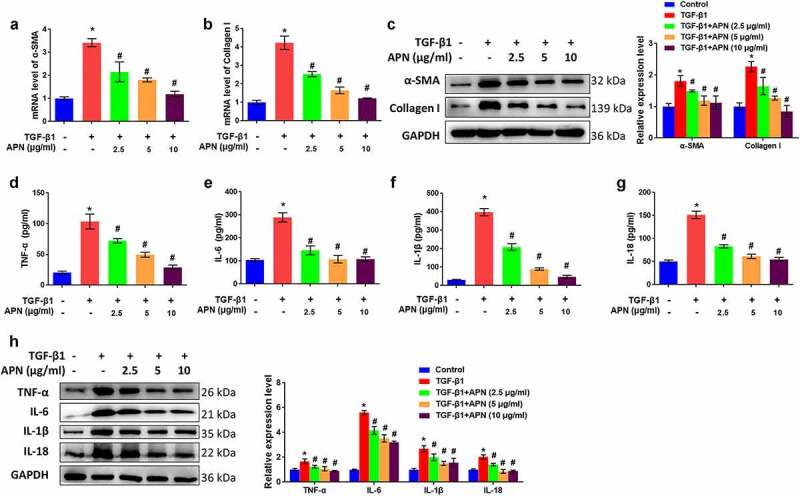
Effects of APN on fibrosis and inflammation in HELF cells. HELF cells were treated by TGF-β1 and different concentrations of APN. mRNA levels of (a) α-SMA, and (b) collagen I in cell were tested by qRT-PCR. (c) The protein levels of α-SMA and collagen I were tested by western blotting. Levels of (d) TNF-α, (e) IL-6, (f) IL-1β, and (g) IL-18 in cell were measured by ELISA. (h) The protein expression of cytokines was measured by western blotting. n = 3. * *p* < 0.05 vs. nontreated group. # *p* < 0.05 vs. TGF-β1-treated group.

The effects of APN on fibrosis-related proteins were studied *in vitro* to further confirm the antifibrotic effect of APN. TGF-β1 significantly elevated mRNA levels of α-SMA and collagen I (*p* < 0.05, Figure 4a,b). APN at different doses (2.5, 5, and 10 μg/ml) decreased mRNA levels of α-SMA and collagen I (*p* < 0.05). Western blot results indicated that protein levels of α-SMA and collagen I were upregulated by TGF-β1 and downregulated by APN (*p* < 0.05, Figure 4c).

The effects of APN on HELF cell inflammation were studied by analyzing the release of pro-inflammatory cytokines in the supernatant. TGF-β1 induced a significant accumulation of TNF-α, IL-6, IL-1β, and IL-18 in the supernatant (*p* < 0.05, Figure 4d-g). APN reduced the concentration of these cytokines in a dose-dependent manner (*p* < 0.05). The protein levels of the four cytokines were remarkably upregulated by TGF-β1 and downregulated by APN (*p* < 0.05, Figure 4h). Taken together, these data suggest the potential of APN to inhibit TGF-β1-induced fibrosis and inflammation in HELF cells.

### APN inhibits lung fibroblast activation by regulating the NF-κB pathway

**Figure 5.**
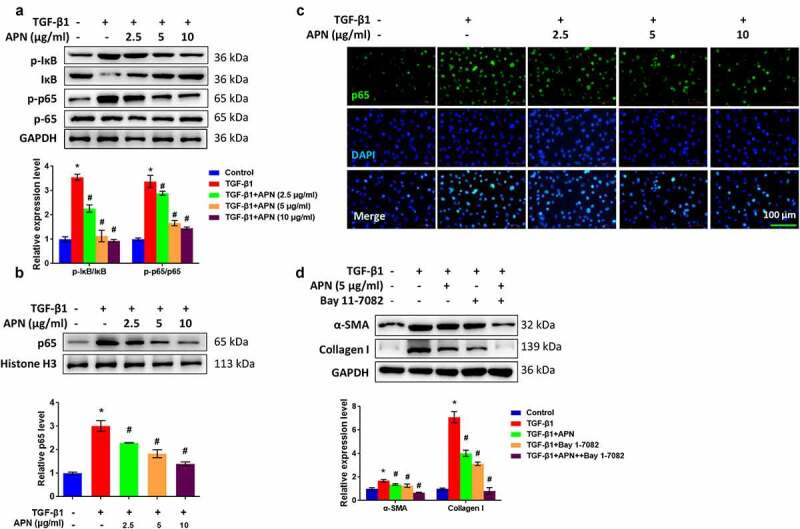
Effects of APN on regulating the NF-κB signaling pathway. HELF cells were treated by TGF-β1 and different concentrations of APN. (a) IκB and p65 in whole cell lysate, and (b) p65 in nuclei were analyzed by western blotting. (c) The level of p65 in nuclei was tested by immunostaining. (d) Bay 11–7082 was used to treat cells for 1 h and then western blotting was performed to detect fibrosis-related proteins. n = 3. * *p* < 0.05 vs. nontreated group. # *p* < 0.05 vs. TGF-β1-treated group.

To reveal the underlying mechanism of which APN exerts its antifibrotic effect, the role of APN in the activation of the NF-κB signaling pathway was studied *in vitro*. To this end, the main manifestations of canonical NF-κB signaling pathway, including IκB degradation and the nuclear translocation of p65 were examined. Figure 5a shows that the phosphorylation of IκB and p65 in the whole-cell lysate TGF-β1-treated cells was significantly increased (*p* < 0.05). The p65 level in the nucleus was increased by TGF-β1 (Figure 5b,c), as detected by western blot analysis and immunofluorescence. The above-mentioned changes induced by TGF-β1 were attenuated by APN (*p* < 0.05), indicating that APN inhibited NF-κB activity by controlling the nuclear translocation of p65. Furthermore, the cells were treated with Bay 11–7082, an inhibitor of NF-κB signaling to investigate whether APN exerted its antifibrotic effects by regulating the NF-κB signaling. As shown in Figure 5d, Bay 11–7082 exerted inhibitory effects on α-SMA and collagen I protein expression, which was similar to the effects of 5 μg/ml of APN (*p* < 0.05). Furthermore, the coadministration of Bay 11–7082 and APN further inhibited α-SMA and collagen I expression (*p* < 0.05). These data indicate that APN inhibited lung fibroblast activation, possibly by regulating the NF-κB pathway.

## Discussion

Antifibrotic agents are currently considered as effective therapies for delaying the progression of IPF and prolonging the survival of patients with IPF [4]. In this study, both *in vivo* and *in vitro* experiments were conducted to reveal antifibrotic effects of APN on IPF, suggesting APN as a potential antifibrotic agent. APN significantly attenuated BLM-induced body weight loss, alveolar destruction, collagen fiber accumulation, and alveolar inflammation. In addition, APN significantly attenuated TGF-β1-induced proliferation, inflammation, and fibrosis in HELF cells. Furthermore, we revealed that APN exerted antifibrotic effects possibly through regulating the activation of the NF-κB signaling pathway. These results confirmed the involvement of APN in the progression of IPF. The effect of APN on pulmonary fibrosis has been previously investigated in paraquat-treated mice [16], whereas we used BLM to develop an IPF animal model, because the BLM model can recapitulate many features of IPF with good reproducibility [35].

Excessive proliferation and activation of human lung fibroblasts is one of the main causes of IPF [36]. Activated lung fibroblasts produce α-SMA and collagen I to destroy the structure of alveoli and induce irreversible fibrosis. Inhibition of fibroblast proliferation and activation has been considered as an effective strategy to control fibrotic progression in IPF [37]. In the current study, HELF cells were treated with TGF-β1, a mediator of fibroblast activation, to induce excessive proliferation. APN significantly inhibited TGF-β1-induced HELF cell proliferation, as evidenced by the downregulation of PCNA and upregulation of p21 and p27. These results confirmed the anti-proliferative effects of APN on lung fibroblasts. The inhibitory effect of APN on the proliferation of other types of fibroblasts has been reported previously, such as keloid fibroblasts [38] and adventitial fibroblasts [39,40]. However, we revealed for the first time the anti-proliferative function of APN in lung fibroblasts.

Chronic alveolar inflammation and interstitial fibrosis are the two main pathological features of IPF. The basis of chronic inflammatory response in the lung is multifactorial, and TGF-β1 signaling is believed to be one of the key pathways involved [41]. Some studies have utilized TGF-β1 and BLM to induce initial inflammation in cell or mouse models of IPF [42,43]. In this study, APN was found to be effective in inhibiting TGF-β1- and BLM-induced inflammation, as indicated by a significant downregulation of related cytokines (TNF-α, IL-6, IL-1β, and IL-18). This result was consistent with a previous study in mice infected with *Aspergillus fumigatus* Af293 conidia [44], revealing the anti-inflammatory function of APN in lung tissues.

NF-κB signaling has been widely accepted as a key pathway involved in regulating cell proliferation, inflammation, and immune responses [18]. Under stimulation, IκB is phosphorylated, which induces its degradation. NF-κB released from IκB translocates into the nucleus and mediates the transcription and translation of target genes [45]. In the present study, p65 was found to be highly expressed in the lung tissues of BLM-treated mice. Moreover, NF-κB signaling can be enhanced by TGF-β1, and thus induce the release of pro-inflammatory cytokines [46,47], indicating the involvement of NF-κB signaling in IPF. Further investigation revealed that APN significantly inhibited the activation of NF-κB signaling pathway, as the phosphorylation of IκB and nuclear translocation of p65 was impeded. Previous studies have revealed the inhibitory effects of APN on NF-κB signaling in various diseases, such as Alzheimer’s disease [24], atherosclerosis [48], osteoporosis [49], and acute pancreatitis [50]. We suggest for the first time that NF-κB signaling is a key pathway involved in the antifibrotic effects of APN in response to BLM stimulation.

There are some limitations to the present study. 1) Considering the wide range of biological functions of APN, APN exerts its antifibrotic effects possibly via a multi-level and multi-target approach. Further studies on the targets of APN should be conducted to fully understand its complexity. 2) More studies should focus on investigating whether chronic doses and systemic delivery of APN lead to side effects. 3) APN delivery strategies, such as nanoparticle-based sustained drug delivery, remain to be studied. 4) The protective effects of APN in mice under different routes of administration and loading dose of BLM need to be confirmed.

## Conclusion

In conclusion, this study provides *in vivo* and *in vitro* evidence of the antifibrotic effects of APN. APN inhibit the proliferation, activation, and inflammation of lung fibroblasts, which controls lung fibrosis. Furthermore, we revealed that APN inhibits the progression of IPF through the NF-κB signaling pathway. These results suggest that APN is an effective therapy for IPF.

## Data Availability

The datasets used and analyzed during the current study are available from the corresponding author on reasonable request.
